# Radiosensitizer EXO-miR-197-3p Inhibits Nasopharyngeal Carcinoma Progression and Radioresistance by Regulating the AKT/mTOR Axis and HSPA5-mediated Autophagy

**DOI:** 10.7150/ijbs.69934

**Published:** 2022-02-21

**Authors:** Jiahui Jiang, Qiao Tang, Jiaoe Gong, WeiHong Jiang, Yiting Chen, Qi Zhou, Alaa Aldeen, Shuanglian Wang, Chengmin Li, Wuwu Lv, Tao Du, Xingwei Wang, Xueying Long, Xueping Feng

**Affiliations:** 1 Department of Otolaryngology-head and Neck Surgery, Department of Oncology and Institute of Medical Sciences, National Clinical Research Center for Geriatric Diseases, Xiangya Hospital, Central South University, Changsha, Hunan Province 410008, China.; 2 Department of Urology and Institute of Medical Sciences, National Clinical Research Center for Geriatric Diseases, Xiangya Hospital, Central South University, Changsha, Hunan Province 410008, China.; 3 Hunan Children's Hospital, Changsha, Hunan Province 410008, China.; 4 Department of Pathology and Institute of Medical Sciences, National Clinical Research Center for Geriatric Diseases, Xiangya Hospital, Central South University, Changsha, Hunan Province 410008, China.; 5 Department of Oncology, The Fourth Affiliated Hospital, Zhejiang University, Yiwu, Zhejiang Province 322000, China.; 6 Department of Histology and Embryology, Xiangya School of Medicine, Central South University, Changsha 410013, China; 7 Department of Radiology, Xiangya Hospital, Central South University, Changsha, Hunan Province 410008, China.

**Keywords:** Nasopharyngeal carcinoma, Exosomes, MicroRNA, HSPA5, Radioresistant cells

## Abstract

The biological functions of exosomes and microRNAs (miRs) in nasopharyngeal carcinoma (NPC) remain largely unexplored. Here, miR-197-3p was screened and identified, and whose level was reduced in serum and exosomes of patients with NPC. MiR-197-3p might be a good diagnostic and prognostic indicator. Our data showed that miR-197-3p expression was closely related to radioresistance, apoptosis, proliferation, migration, and survival of NPC. Inhibition of miR-197-3p expression *in vitro* could promote the proliferation and migration of NPC cells, while promotion of miR-197-3p expression *in vivo* could significantly inhibit the growth and enhance the radiosensitivity of NPC cells. From the perspective of mechanism, miR-197-3p could inhibit AKT/mTOR phosphorylation activation, inhibit an activated pathway of AKT/mTOR, target Heat Shock 70-kDa Protein 5(HSPA5) related to endoplasmic reticulum homeostasis, inhibit HSPA5-mediated autophagy, and reverse the radioresistance of NPC. Interestingly, exosomal miR-197-3p (EXO-miR-197-3p) reduced the proliferation and migration potential of NPC cells *in vitro*, and tumor growth and radioresistance of NPC cells *in vivo*. EXO-miR-197-3p inhibited NPC progression and radioresistance by regulating AKT/mTOR phosphorylation activation and HSPA5-mediated autophagy. In conclusion, our results highlight the potential of EXO-miR-197-3p as an effective radiosensitizer and therapeutic agent for refractory NPC.

## Introduction

Nasopharyngeal carcinoma (NPC), associated with Epstein-Barr virus (EBV) infection, is the most common malignant lesion of the nasopharynx. NPC arises from the nasopharyngeal mucosa, and exhibits distinct regional and racial prevalence, occurring most frequently in the east and southeast Asia 1. Ionizing radiation (IR) is the most effective treatment for patients with early stage NPC receiving local radiotherapy 2, 3. Intensity-modulated radiation therapy for NPC can decrease the radiation dose and has demonstrated encouraging clinical outcomes 4, 5. It was reported that 30-40% of patients with NPCs were radioresistant, who were refractory NPCs for progressive diseases despite intensive treatment 6. The radioresistance of NPC remains a bottleneck in clinical therapy because of recurrence and distant metastasis 7. It remains a major challenge for the treatment of patients with relapsed and refractory NPC, which will result in low overall survival (OS) 8.

Over the past 30 years, several breakthroughs have been made in molecular medicine 9. However, refractory NPC cells display the radioresistance related to cellular radiation response. The radiation response of cancer cells is complex, which is often determined by the intrinsic ability of gene repair. Exosomes can transfer functional molecules, including microRNAs (miRNAs, miRs) and proteins, into recipient cells, which is involved in intercellular communication of cancer cells. In the tumour microenvironment, cells experience nutrient deprivation, hypoxia, or drug-induced toxicity, all of which interfere with protein folding. An increasing number of misfolded proteins accumulate in the endoplasmic reticulum (ER), leading to a phenomenon called ER stress 10. Several reports have shown that inhibiting the production of cancer-derived exosomes may exert therapeutic effects by inhibiting cancer proliferation and dissemination. However, the biological functions of exosomes and miRNAs remain largely unexplored, specifically about NPC radioresistance.

Recent studies have shown that miRNAs play an important role in the proliferation, migration, invasion, and radioresistance of NPC 11. Specifically, miR-197-3p level is downregulated in a variety of tumors 12-15. The downregulated miR-197-3p may be a predictor of poor prognosis in patients with hepatocellular carcinoma 12. Previous studies have shown that miR-197-3p can regulate the occurrence and development of tumors through the AKT pathway, and inhibit the growth of xenogeneic tumors *in vivo* 13. In addition, miR-197-3p can inhibit the proliferation, migration, and invasion of some tumor cells related to the inhibition of epithelial mesenchymal transformation (EMT) 14, 15. In fibrosarcoma cells, miR-197-3p can also trigger G2/M phase cell cycle arrest and induce autophagy 16. Presently, the role of miR-197-3p in NPC has not yet been reported.

The heat shock protein family A (Hsp70) member 5 (HSPA5) is a multifunctional protein related to endoplasmic reticulum homeostasis 17. HSPA5 can increase ionizing radiation resistance in NPC, but the specific molecular mechanism remains to be elucidated 18. In our previous study, HSPA5 was found to be upregulated in radioresistant NPC cells 19-21. HSPA5 was a prognosis biomarker of NPC 20, 21, which played an important role including the regulation of various biological processes such as EMT 19-21. Studies have shown that cell surface HSPA5 is an upstream regulator of the PI3K/AKT signalling pathway by targeting HSPA5 to inhibit tumor progression 22. In addition, after anti-HSPA5 treatment, the PI3K/Akt/mTOR signalling pathway was inhibited, cell proliferation and colony formation weakened, and apoptosis enhanced. Radiotherapy combined with the treatment of anti-HSPA5 resulted in significant tumor growth delay, and enhanced radiotherapy efficacy in glioblastoma and non-small cell lung cancer 23.

Furthermore, it was reported that autophagy was activated in NPC after radiotherapy, which promoted radioresistance 24. The radioresistant NPC cell lines had an increased incidence of autophagy *in vitro*, and LC3 silencing could make NPC cells sensitive to radiation 25. In addition, inhibition of autophagy and activation of the caspase system can significantly enhance the radiosensitivity of NPC cells 26-28. Therefore, inhibition of autophagy may provide a new treatment option for radioresistant NPC 29. In oropharyngeal cancer cells, silencing of HSPA5 has been shown to increase radiosensitivity and inhibit autophagy 30, 31.

As discussed above, exosomes enriched with miR-197-3p (referred to hereafter as EXOs-miR-197-3p) may provide a novel strategy for the treatment of radioresistant NPC. It is of great significance to study the mechanistic details associated with EXO-miR-197-3p action, which appears to be mediated by AKT/mTOR regulation and HSPA5-mediated autophagy. EXOs-miR-197-3p might have the potential to inhibit NPC progression and radioresistance.

In this study, our results suggested that EXOs-miR-197-3p had a function of HSPA5 silencing, which increased radiosensitivity and inhibited autophagy of NPC cells. We will study its mechanism in more detail.

## Materials and Methods

### Data collection preprocessing

The Starbase website (https://starbase.sysu.edu.cn/) was used to identify differences in target genes between NPC and normal tissues. Kaplan-Meier Plotter database (https://kmplot.com/analysis/) was used for survival analysis.

### Patient samples

Between 2019 and 2021, 40 newly NPC tissue biopsies of total 432 patients and 21 chronic inflammation of nasopharyngeal mucosa tissue biopsies from patients receiving pharyngorhinoscopy inspection were collected at Xiangya Hospital, Central South University. The 40 NPC patients were treated with the total dosages of radiotherapy for 68 ~72 Gy (2 Gy/fraction, 5 days/week, followed by rest for 2 days), who did not receive other anticancer treatments before and after the biopsies. The therapeutic effects were evaluated by magnetic resonance imaging (MRI) after 3 months of radiotherapy. Based on MRI images of the tumors before and after treatment, complete response (CR) patients were defined as radiosensitive groups, while stable disease (SD) and progressed disease (PD) patients were defined as radioresistance groups. Radioresistant NPC patients were defined as patients with local recurrence at nasopharynx and/or cervical lymph nodes ≤12 months after completion of radiotherapy. Radiosensitive NPC patients were defined as patients without local residual lesions at > 3 months and without local recurrence > 12 months after the end of radiotherapy. All samples were diagnosed by two or more experienced pathologists. Then the NPC patients were divided into two groups. The radiotherapy sensitive group showed complete remission of NPC tumor after radiotherapy, which suggested that the NPC patients had relatively good radiotherapy effects (20 cases). The radioresistance group consisted of NPC patients with stable progression of cancer after radiotherapy, which suggested that the NPC patients had relatively poor radiotherapy effects (20 cases). Details of patients are shown in Table S1.

Serum samples from healthy people were collected in the Health Management Center of Xiangya Hospital, Central South University. All patients provided written informed consent, and the Research Ethics Committee of Xiangya Hospital, Central South University approved this study, which was consistent with the Declaration of Helsinki.

### Immunohistochemistry (IHC) staining

Paraffin-embedded tissue sections were heated at 65 °C for 1h. The sections were dewaxed by Xylene, and hydrated using gradient ethanol. The samples were immersed in boiling EDTA- antigen repair solution and heated in a microwave oven (95-100 °C for 20 min). The samples were treated with 3% H_2_O_2_ at room temperature for 10 min to block endogenous peroxidase activity, PBS 3 times, then added 10% goat serum for 1 hour. Corresponding primary antibodies at appropriate concentrations were incubated at 37 °C for 1 h. After PBS was properly cleaned, the corresponding secondary antibodies were incubated for 30 minutes; PBS 3times, added 3, 3-diaminobenzidine (DAB) for color rendering, and hematoxylin was counter-stained for 3 minutes. In negative control staining, PBS instead of primary antibody. The samples were evaluated according to the intensity and degree of staining.

### Cell culture

The NPC cell lines C666-1 (human, XY-XB-3163) and HK-1 (human, SUER0025) were purchased from the Cell Bank of the Type Culture Collection of the Chinese Academy of Sciences (Shanghai, China). The radiation-resistant C666-1 cell line (C666-1R) and HK-1 cell line (HK-1R) was established by Prof. Feng 19-21, Xiangya Hospital of Central South University, China. The parent cells were irradiated at dosages of 2Gy, 4Gy, 6Gy, 8Gy. The stable radioresistant cells were formed after a total dosage of 40Gy. All cells were cultured in RPMI-1640 medium (Biological Industries, 01-100-1A) containing 10% fetal bovine serum (FBS) (Biological Industries, 04-001-1A) at 37 °C with 5% CO_2_.

### Plate colony formation assay

A certain number of NPC cells were cultured in six-well plates. After cell adherence, transfection or EXO co-culture was performed. The cells were irradiated with X-rays (4Gy or 8Gy) and dosimetry confirmed the central dose rate of 100 cGy/min (X-RAD 225 high-energy biological X-ray irradiator, USA), then further incubated at 37 °C with 5% CO_2_ for 2-3 weeks. After cell colonies (50 cells) were formed in the six-well plate, the cells were washed twice with phosphate buffered saline (PBS) (Solarbio, P1020), fixed with 4% Paraformaldehyde Fix Solution (Beyotime, P0099) for 10 min, cleaned 2 times with PBS, stained with 0.1% crystal violet (Beyotime, C0121) at 37 °C for 10 min, followed by PBS cleaning twice, and photographed after drying.

### Cell counting kit-8 (CCK-8) assay

Cell proliferation was detected by CCK-8. The cells were seeded into 96 well plates with 5×10^3^ cells in each well. After incubation at 37 °C with 5% CO2 for an appropriate length of time (after adherence, day 3, day 5), the cells were incubated with 10 μL CCK-8 solution (APE×BIO, K1018) for 2 h. The absorbance at 450nm was then measured with a multifunctional microplate meter (TECAN, Austria).

### Quantitative real-time PCR (qRT-PCR)

The primer sequences are as follows: miR-197-3p: 5' -UUCACCACCUUCUCCACCCAGC-3'; Quantitative real-time PCR (qRT-PCR) was used to analyze the expression of miR-197-3p in cells of each group. Total RNA was extracted from cells using Trizol reagent (Invitrogen, A33250) and then reversely transcribed into cDNA using a reverse transcription kit (Takara, 638315). Then, qPCR SYBR Green Master Mix (YEASEN, 11202ES08) was used for qRT-PCR analysis. The relative expression of miR-197-3p was calculated by 2^-ΔΔCt^ method.

### Western blot

After the total proteins of cells in each group were isolated with total protein extraction kit (Transgen, DE101-01), protein samples were treated with SDS-PAGE protein loading buffer (Beyotime, P0015). Then the cell proteins were separated by 10% sodium dodecyl sulfate polyacrylamide gel electrophoresis, which were transferred to a polyvinylidene fluoride membrane (Millipore), washed with TBST buffer (Solarbio, T1081). The membrane was sealed with 5% skim milk for 1h, and incubated with primary antibody at 4 °C overnight, and the second antibody incubated at 37 °C for 1h. Then, the samples were washed 3 times with TBST buffer (Solarbio, T1081), and ECL luminescence solution (Biosharp, BL520A) was added. The protein bands were observed using a chemiluminescence gel imaging system (Bio-RAD, USA). The used antibodies are as follows: GAPDH (Transgen, HC301-01), HSPA5 (Proteintect, 11587-1-AP), LC3B (Abcam, ab192890), P62 (BOSTER, M00300-1), Phospho-mTOR (Ser2448) (D9C2) (Cell Signaling Technology, 5536T), mTOR (7C10) (Cell Signaling Technology, 2983T), Phospho-Akt (Ser473) (D9E) (Cell Signaling Technology, 4060S), Akt (pan) (C67E7) (Cell Signaling Technology, 4691S), Exosome Panel (Calnexin, CD9, CD63, CD81, Hsp70, TSG101) (ab275018), Goat Anti-mouse IgG (Transgen, HS201-01), Goat anti-rabbit IgG (Transgen, HS101-01).

### Cell transfection and lentivirus infection

NPC cells were transfected with Lipofectamine 3000 (Invitrogen, L3000015). Mir-197-3p mimic and negative control (mimic NC), miR-197-3p inhibitor and negative control (inhibitor NC), GFP-LC3 plasmid and HSPA5 Vectors, pcDNA3.1 were obtained from Shanghai Sangon Biotechnology Company, China. Lv-hsa-mir-197 was purchased from Genechem Company (Shanghai, CHINA). Follow instructions for lentivirus infection. (MOI=10). After the cells were adhered to the wall, the above substances were transfected into the cells. After transfection for 48 h, the cells were collected, used and/or stored.

### Cell immunofluorescence

NPC cells were cultured in a six-well plate with glass slides. The cells were fixed with 4% Paraformaldehyde Fix Solution for 1h after adherence. After washing with PBS, the non-specific binding sites were sealed with goat serum at room temperature for 30 min. After absorbing goat serum with filter paper, the cells were incubated with primary antibodies at 4 °C overnight, washed with PBS for 3 times at the next day, and incubated with the secondary antibody at room temperature in darkness for 1 h, washed with PBS, stained with DAPI (Solarbio, C0065) for 3 min, and again washed with PBS. The anti-fluorescence quenching sealed tablet was then applied (Servicebio, G1401). Imaging was performed with a confocal laser microscope (Leica, Germany).

### EdU assay

EdU detection was performed using the EdU detection kit (RiboBio, C10310-3). The NPC cells were cultured in 96-well plates (10^4^ cells/well) to the normal growth stage and treated accordingly. Incubate EdU (50 µM) for 2 hours, and cleaned with PBS. After fixation with 4% paraformaldehyde for 30 minutes, the NPC cells was cleaned with PBS, and subsequently incubated with 0.5% TritonX-100 (Solarbio, T8200) for 10 min, then incubated and treated according to the instructions. After all the steps were completed, fluorescence imaging was observed using a fluorescence microscope (Leica, Germany).

### Wound healing assay

Cell migration was assessed by wound healing assay. The treated NPC cells were seeded into 6-well plates, which were cultured until 90% confluence. Scratches of cells were made with a 200-μl sterile pipette tip, and further grown in a 5% serum medium after the exfoliated cells were washed with PBS. The wound healing, 72 h later, was observed and imaged every 12 h under microscope.

### Transwell assay

The Transwell assay was performed using a Costar Transwell culture plate (Corning Incorporated, USA) to evaluate the capability of cell migration *in vitro*. The NPC cells (5×10^4^ cells) suspended in serum-free medium were placed in the upper chamber, which would migrate into the lower chamber with the 20% FBS-containing medium served as the chemoattractant. After 24 h culture, the cells migrated to the lower chamber were fixed with 4% paraformaldehyde. After washed with PBS, the cells was stained with 0.1% crystal violet, and 5 random fields of the cells were counted by inverted microscope.

### Flow cytometry

An apoptosis assay was performed using the Annexin V-FITC/PI Apoptosis Detection Kit (Vazyme, A211) or YF647A-AnnexinV/PI Apoptosis Detection Kit (UElandy, Y6026L) 17. Propidium Iodide was used with Annexin V to determine if cells were viable, apoptotic, or necrotic, which was analyzed by flow cytometry (BD Biosciences, USA) and Flowjo or Kaluza software.

### Animal studies

Male BALB/c nude mice, 4-6 weeks old, were obtained from Hunan SJA Laboratory Animal Company. The experiments were approved by Institutional Animal Care and Use Committee of Central South University. For tumor growth assay, the treated NPC cells were injected subcutaneously into the dorsal flank of nude mice (10^7^ cells per mouse, five mice per group). Tumor volume was measured every three days beginning on day 6. The tumors 15 days later were locally irradiated with 4Gy. Tumor measurements and experiments continued.

### Dual Luciferase reporter assay

The relationship between HSPA5 and miR-197-3p was determined using the dual luciferase reporter assay. HSPA5 containing the predicted binding site (HSPA5-WT) or mutant binding site (HSPA5-Mut) was amplified and cloned into the pSI-Check2 vector (Hanbio, China). The miR-197-3p mimic, NC mimic, and plasmid were co-transfected into HEK-293T cells. At 48 h after transfection of the pSI-Check2 vector, the luciferase activity was measured using the dual luciferase reporter gene assay system (Promega, E1910).

### Exosome isolation and observation of exosome labeling

Exosomes were isolated from cell culture medium by ultracentrifugation (Beckman. USA) and ExoQuick-TC Exosome Isolation Reagent (SBI, EXOTC10A-1). The collected cell culture supernatants were first centrifuged at 2000×g, then which were filtered and ultracentrifuged at 110,000×g for 2 h. Exosomes were collected from the pellets resuspended in PBS.

The samples were observed with a transmission electron microscope by Department of Electron Microscopy, Xiangya Medical College, Central South University. In the co-location experiment, according to the instructions, exosomes were stained with PKH26 purchased from Sigma-Aldrich (Shanghai) Trading Co. Ltd for follow-up observation.

## Results

### HSPA5-associated radioresistance of NPC

**Figure 1:**
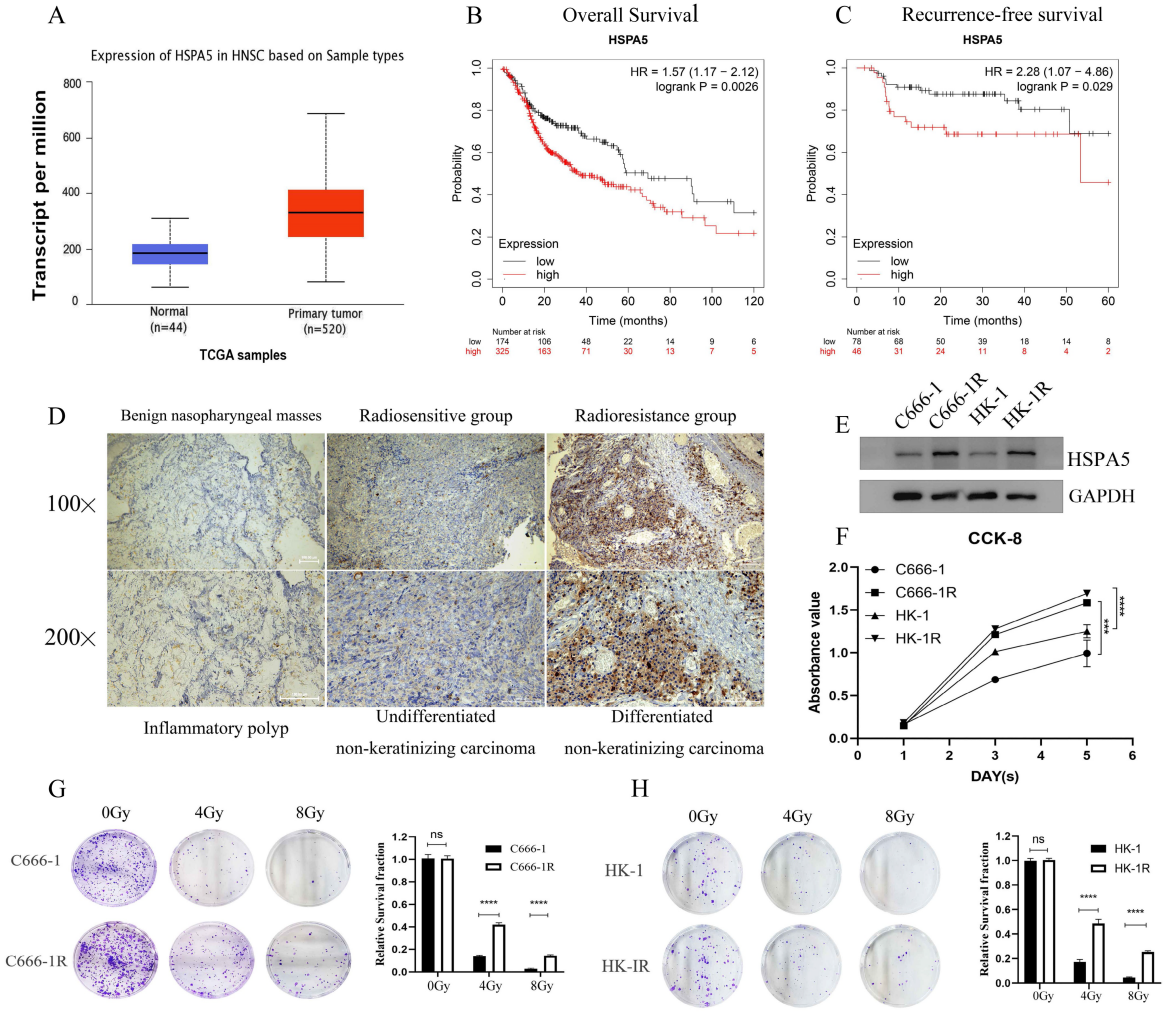
** HSPA5-associated radioresistance of NPC. (A)** Starbase database analysis showed that the expression of HSPA5 was correlated with the occurrence of squamous cell carcinoma of the head and neck. **(B, C)** Kaplan-Meier Plotter database analysis showed that the expression of HSPA5 was correlated with the prognosis of squamous cell carcinoma of the head and neck. **(D)** Immunohistochemical analysis was performed to analyze the correlation between HSPA5 and radioresistance of NPC. The expression of HSPA5 was increased in NPC tissues compared to benign nasopharyngeal masses; HSPA5 was expressed more higher in the recurrent radioresistant NPC tissues than in the radiosensitivity NPC tissues. **(E)** Western Blot experiment verified the difference for HSPA5 higher in radioresistant NPC cells than in the parent cells. **(F)** The CCK-8 assay showed the difference in proliferation between the parental and constructed cell lines; and the proliferation ability of the radioresistant NPC cell lines was found to be greater.** (G, H)** Colony formation experiments were performed to verify the differences in colony formation ability upon treatment with radiation; and the radioresistance of C666-1R and HK-1R was significantly enhanced in comparison to the parental cell lines.

In the previous stage, our research group conducted a study on the correlation between the expression of HSPA5 (GRP78) and the radioresistance of NPC 19-21. According to analysis of Starbase website and Kaplan-Meier Plotter database, the expression of HSPA5 was significantly correlated with the occurrence of Head-neck squamous cell carcinoma (HNSC) (Figure 1A) and prognosis (Figure 1B, 1C). To further analyze the correlation between HSPA5 and radioresistance of NPC, immunohistochemical analysis was performed. All patients with NPC (including patients with recurrent NPC) admitted to Xiangya Hospital of Central South University in recent 3-year period (2019-2021) were selected for analysis of radioresistance. Among them, a total of 40 patients with NPC (including 20 patients with recurrent NPC) were selected to analyze the expression of HSPA5 in tumors (Table S1). In addition, pathological samples of 21 benign nasopharyngeal masses were collected as controls (Table 1). Our results showed that the expression of HSPA5 was increased in NPC tissues compared to benign nasopharyngeal masses; HSPA5 was expressed more obviously in the recurrent radioresistant NPC tissues compared to the radiosensitivity NPC. (Figure 1D; Table 2). The constructed cell line C666-1R and HK-1R by our research group (Materials and Methods) were selected for corresponding verification. Western Blot experiment verified the difference of HSPA5 expression in radioresistant NPC cells (Figure 1E). The CCK-8 assay was used to detect the difference for proliferation of the parental and constructed cell lines. The proliferation ability of the radioresistant NPC cell lines was found to be greater (Figure 1F). At the same time, colony formation experiments were performed to verify the differences in colony formation ability upon cells treated with radiation. The radioresistance of C666-1R and HK-1R was significantly enhanced in comparison to the parental cell lines (Figure 1G, 1H).

### Downregulation of serum exosomal miR-197-3p associated with poor prognosis in radioresistant NPC patients

**Figure 2:**
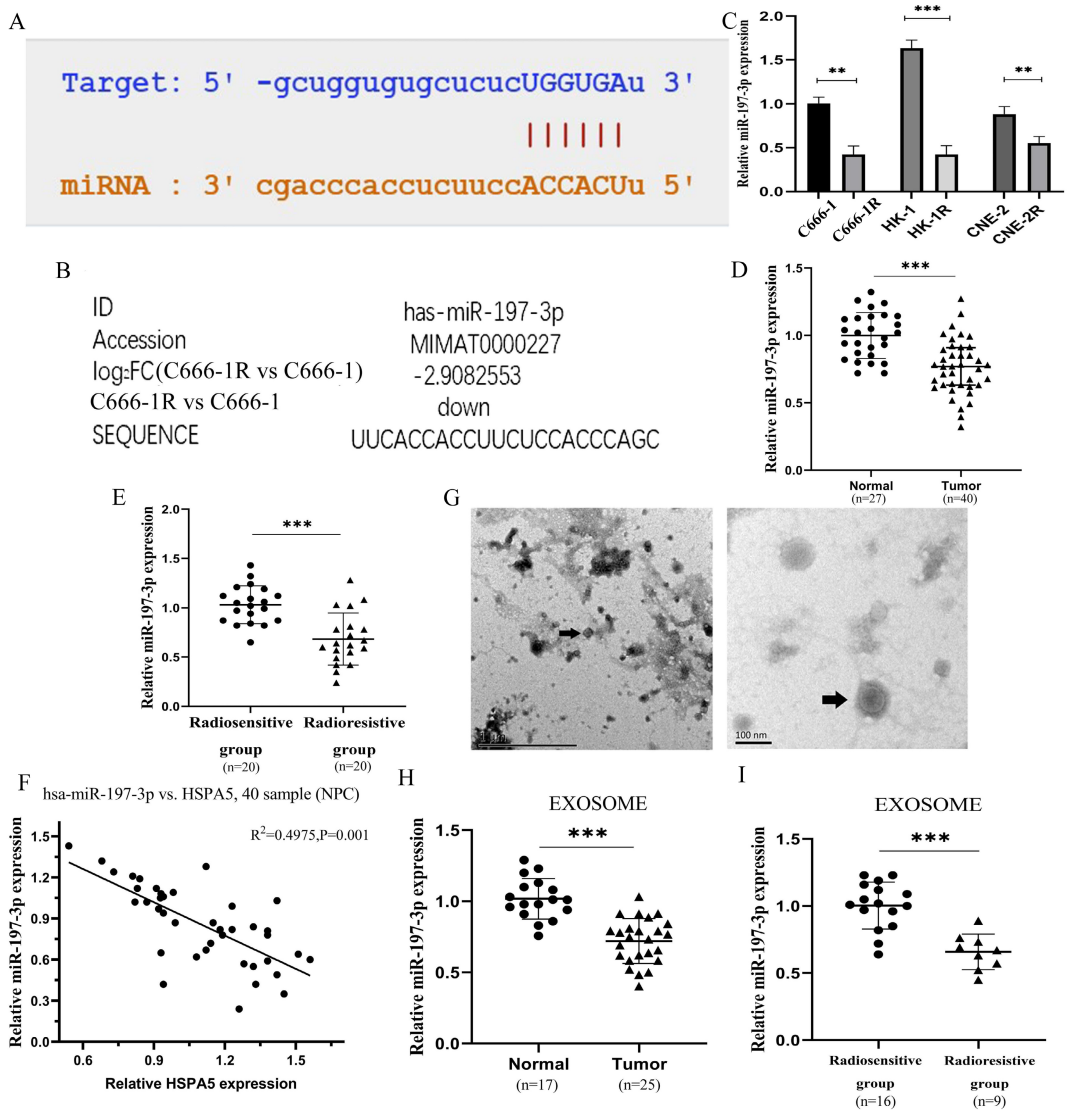
** Downregulation of serum exosomal miR-197-3p associated with poor prognosis in radioresistant NPC patients. (A)** The binding sites of HSPA5 and miR-197-3p were predicted. **(B)** MiR-197-3p was reduced 2.9 times for the radioresistant C666-1R vs the parent control C666-1 by analysis of miRNA microarray. **(C)** The expression of miR-197-3p was identified lower in the multiple radioresistant NPC lines compared to their control lines by qRT-PCR. **(D, E)** The expression of miR-197-3p was analyzed in serum of 27 healthy people and 40 NPC patients by qRT-PCR. **(F)** There was a correlation between HSPA5 and miR-197-3p in NPC samples. **(G)** The characteristics of exosomes were evaluated by transmission electron microscopy. **(H, I)** The expression of exosomal miR-197-3p was higher in healthy people (17 cases) than in NPC patients (25 cases).

The miR-197-3p related to HSPA5 was screened through a variety of prediction websites (Figure 2A), which was also screened from the miRNA microarray analysis of C666-1 vs C666-1R (Figure 2B). The expression of miR-197-3p was identified lower in the multiple radioresistant NPC lines compared to their control lines by qRT-PCR (Figure 2C) 17. MiR-197-3p levels in the radioresistant strains of NPC were reduced, to varying extents, which is consistent with our chip results. A further experiment, the expression of miR-197-3p was analyzed in serum of 27 healthy people and 40 NPC patients by qRT-PCR. Compared with healthy people, the expression of miR-197-3p in NPC patients was significantly decreased (Figure 2D). Subsequently, miR-197-3p was analyzed on the expression of the radiosensitive group (n=20) and the radioresistant group (n=20), indicating that miR-197-3p decreased most significantly in the radioresistant group. It is suggested that miR-197-3p is correlated with NPC, especially radioresistance (Figure 2E). In addition, there was a significant negative correlation with the results of HSPA5 immunohistochemistry conducted earlier (Figure 2F). Then we extracted corresponding samples of serum exosomes for further detection. There were 17 healthy human exosomes, 16 radiosensitive exosomes and 9 radioresistant exosomes. The characteristics of exosomes were evaluated by transmission electron microscopy, which revealed the homogeneous morphology of exosomes, with sizes ranging from 50 to 130 nm (Figure 2G). We detected the expression of exosomal miR-197-3p in healthy people (17 cases) and NPC patients (25 cases). Our data suggested that the expression of exosomal miR-197-3p was down-regulated in NPC patients, which might have a certain diagnostic value (Figure 2H). Meanwhile, miR-197-3p decreased more significantly in serum exosomes of NPC patients with radioresistance 9 cases (Figure 2I). It is suggested that exosome miR-197-3p had certain prognostic effect.

### MiR-197-3p/AKT/mTOR axis inhibits cell proliferation and migration, and promotes apoptosis in NPC cells

**Figure 3:**
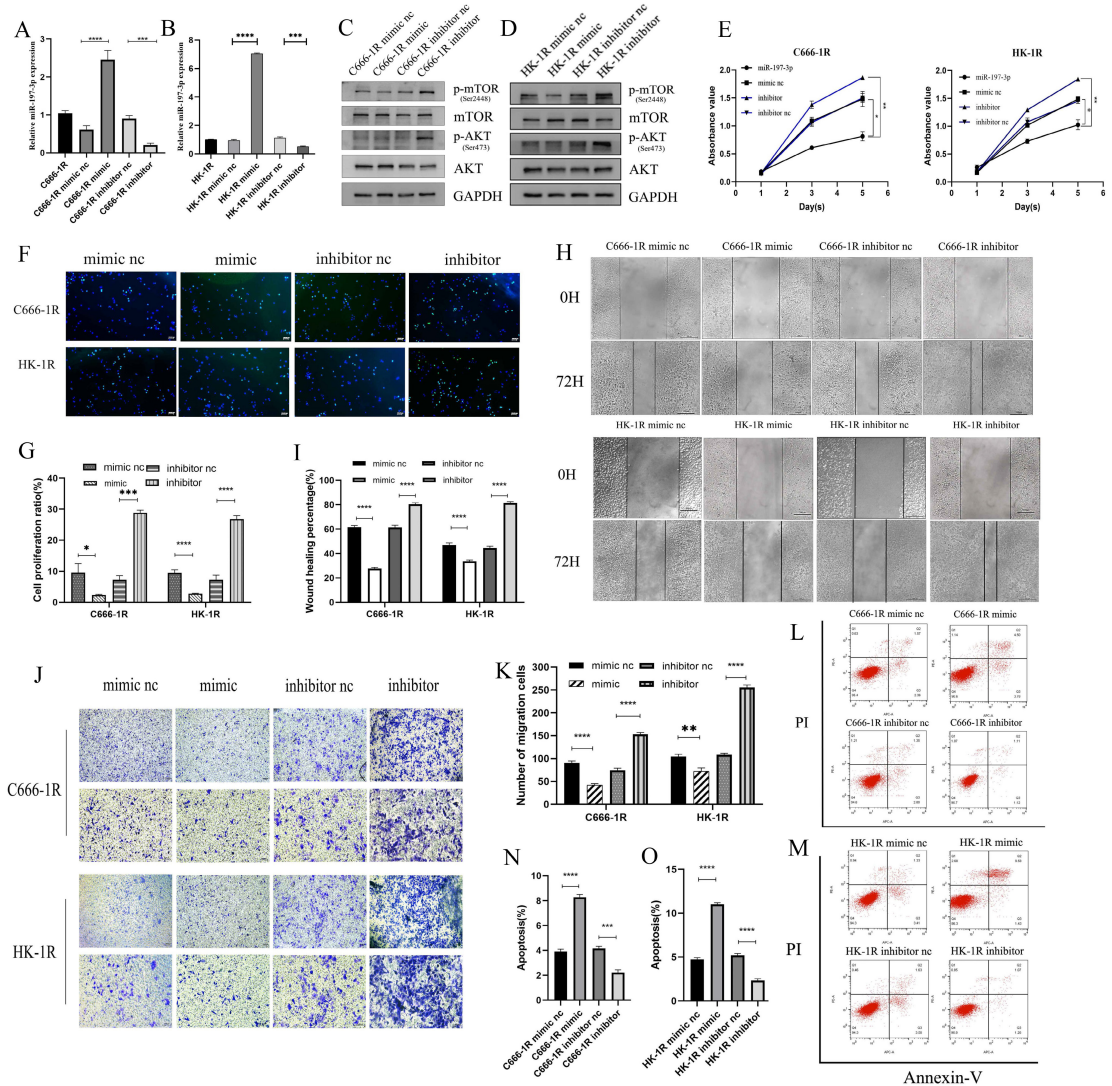
** MiR-197-3p/AKT/mTOR axis inhibits cell proliferation and migration, and promotes apoptosis in NPC cells. (A, B)** QRT-PCR was used to detect the expression of miR-197-3p after transfection. **(C, D)** Western Blot was used to detect the changes of AKT/mTOR pathway after transfection. **(E)** The proliferation ability of transfected cells was detected by CCK-8 assay. **(F, G)** EDU experiment verified cell proliferation changes. **(H, I, J, K)** Wound Healing and Transwell assay verified cell migration. **(L, M, N, O)** Flow cytometry was performed to detect cell apoptosis after tranfection.

Previous studies have shown that miR-197-3p is downregulated in a variety of cancers, inhibits tumor proliferation and invasion, and improves the radiosensitivity of radiation-resistant cells. Previous work in our group has also shown that miR-197-3p is downregulated in radioresistant NPC cell lines, but whether it affects the progression of NPC requires further evaluation. C666-1R and HK-1R cell lines were transfected with miR-197-3p mimic and inhibitor, and the transfection efficiency was verified by qRT-PCR (Figure 3A, 3B). Western blotting detected changes in the transfection pathway, and it was found that the AKT/mTOR pathway was inhibited upon overexpression of miR-197-3p, which might be related to the inhibition of tumor progression (Figure 3C, 3D). The proliferation ability of transfected cells was detected using the CCK-8 assay. Interestingly, overexpression of miR-197-3p decreased cell proliferation, whereas inhibition of miR-197-3p increased cell proliferation (Figure 3E). Meanwhile, the EDU experiment was used to verify changes in cell proliferation (Figure 3F, 3G). Subsequently, we conducted wound healing and transwell assays to verify cell migration, and found that overexpression of miR-197-3p inhibited cell migration, while inhibition of miR-197-3p expression promoted cell migration (Figure 3H, 3I, 3J, 3K). Flow cytometry experiments revealed that miR-197-3p promoted cell apoptosis, while cell apoptosis was reduced after inhibiting the expression of miR-197-3p (Figure 3L, 3M, 3N, 3O). According to the above results, miR-197-3p regulates the AKT/mTOR pathway and affects the progression of NPC.

### MiR-197-3p targeting HSPA5 mediates the effects of NPC radioresistance

**Figure 4:**
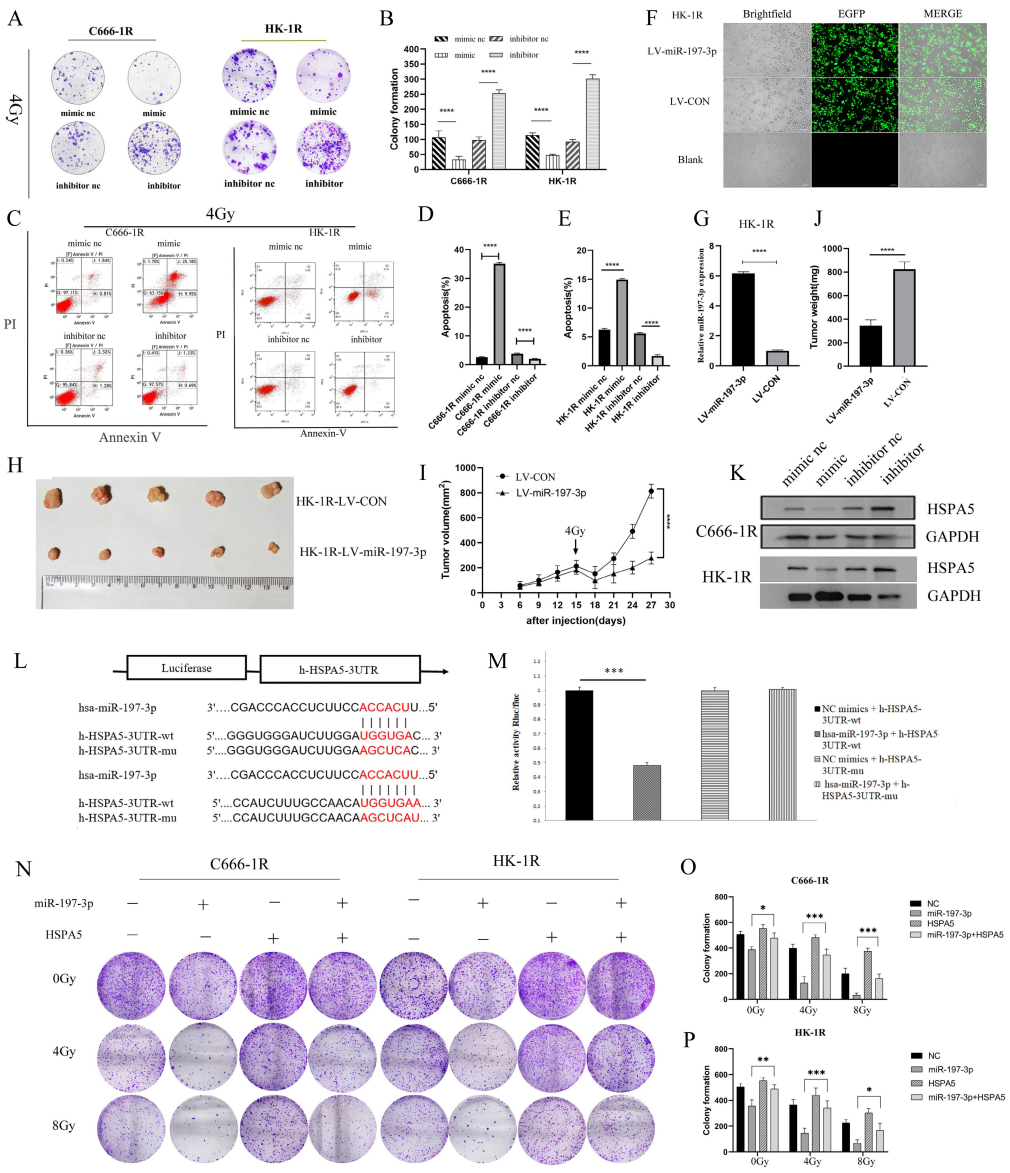
** MiR-197-3p targeting HSPA5 mediates the effects of NPC radioresistance. (A, B)** The radioresistance of transfected cells to 4Gy radiation was observed by colony formation assay. **(C, D, E)** Flow cytometry was used to detect transfected cell apoptosis after 4Gy. **(F)** LV-has-miR-197-3p infected HK-1R cells. **(G)** QRT-PCR detected miR-197-3p expression in HK-1R-LV-miR-197-3p group. **(H, I, J)** Animal experiments have confirmed that miR-197-3p could affect the radiosensitivity of NPC. Tumor volume and weight were monitored. **K.** The protein levels of HSPA5 in transferred NPC cells were detected. **(L, M)** Dual luciferase reporter gene assay verified that miR-197-3p could directly target HSPA5. **(N, O, P)** Colony formation experiments confirmed the regulation of radioresistance of NPC by miR-197-3p targeting HSPA5.

Having successfully verified that miR-197-3p could inhibit the proliferation, migration, and promote apoptosis of NPC cells, we next investigated whether miR-197-3p could regulate the radioresistance of NPC cells. We carried out colony formation experiments to observe the resistance of the transfected cells to radiation. At 4 Gy of irradiation, cells transfected with the mimic were more sensitive to radiation, while cells transfected with the inhibitor were more resistant (Figure 4A, 4B). Flow cytometry was used to detect cell apoptosis after treatment with 4 Gy, and it was found that miR-197-3p significantly reduced cell apoptosis, which might be beneficial to radiotherapy (Figure 4C, 4D, 4E). Subsequently, LV-hsa-mir-197-3p was used to construct the HK-1R cell line with stable overexpression of miR-197-3p (HK-1R-LV-miR-197-3p), and the control group was named HK-1R-LV-CON. Lentivirus was labelled with EGFP and its infection efficiency was observed under a fluorescence microscope, which was close to 100% (Figure 4F), and qRT-PCR detected significantly increased miR-197-3p expression in the HK-1R-LV-miR-197-3p group (Figure 4G). *In vitro*, our study confirmed that miR-197-3p affected the radiosensitivity of NPC. To further study these effects *in vivo*, HK-1R-LV-miR-197-3p was injected into nude mice that were irradiated with 4 Gy on day 15. We found that miR-197-3p inhibited tumor growth, and LV-miR-197-3p tumor volume significantly decreased under irradiation, further supporting the inhibitory effect of miR-197-3p on tumor growth, which was significantly enhanced under irradiation conditions (Figure 4H, 4I, 4J). In addition, we further verified the correlation between miR-197-3p and HSPA5. The expression of miR-197-3p was significantly lower, but the expression of HSPA5 was higher, in radioresistant NPC cells than in the control cells. To further confirm that HSPA5 was downregulated in NPC cells overexpressing miR-197-3p, we measured HSPA5 protein levels in transfected NPC cells. The data showed that, at the protein level, the expression of HSPA5 in NPC cells overexpressing miR-197-3p was significantly lower than that of the control group (Figure 4K). According to a dual luciferase reporter gene experiment, miR-197-3p could directly target HSPA5 (Figure 4L, 4M). We further verified the regulation of radioresistance of NPC by miR-197-3p targeting HSPA5 through colony formation experiments (Figure 4N, 4O, 4P).

### MiR-197-3p downregulating HSPA5 modulates autophagy to control X-ray sensitivity

**Figure 5:**
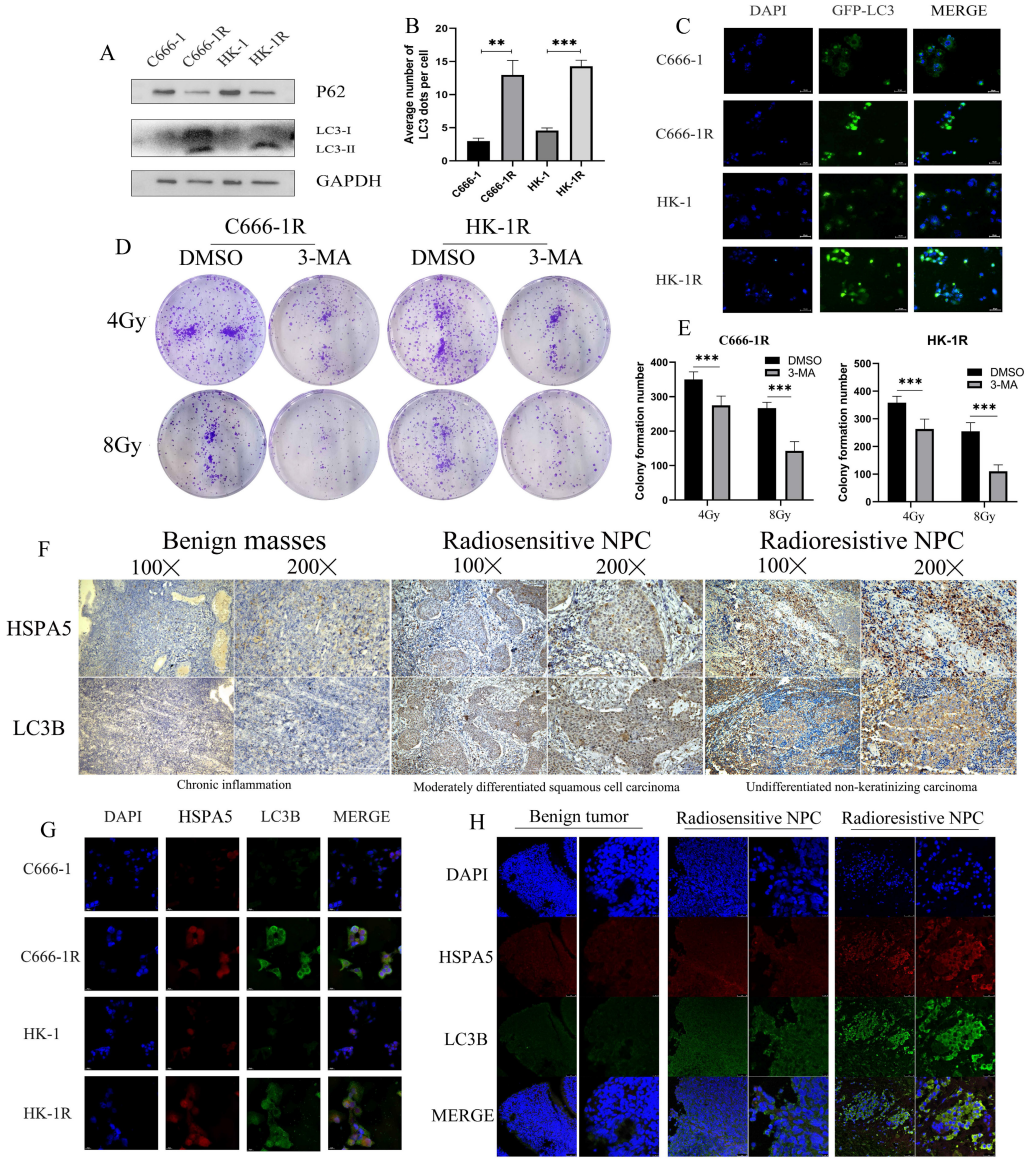
** HSPA5 expression is associated with autophagy in nasopharyngeal carcinoma. (A)** Western blot experiment detected autophagy in NPC cell lines. **(B, C)** GFP-LC3 transfection was used to detect autophagosomes. **(D, E)** Colony formation demonstrated that autophagy inhibitors modulated radiosensitivity of NPC. **(F)** Immunohistochemical experiments showed that the expression of LC3B and HSPA5 in NPC tissues. **(G)** Cell immunofluorescence showed the expression of HSPA5 and LC3B. **(H)** Immunofluorescence showed that the expressions of HSPA5 and LC3B in tumor tissues.

Autophagy is a conservative and necessary process in which the body removes excess cell contents in response to stress, to maintain cell survival 32. Autophagy is thought to contribute to radioresistance in NPC. Western blot experiment was carried out to detect autophagy in cell lines and found that the autophagy of cell lines with radioresistance was greater than the corresponding parental cell lines (Figure 5A). GFP-LC3 was also transfected, and increased autophagosome formation was observed in radioresistant cell lines (Figure 5B, 5C). We further demonstrated *in vitro* that inhibition of autophagy can improve radiotherapy sensitivity. Colony formation showed that the sensitivity of radioresistant NPC cells to radiation increased with the use of the autophagy inhibitor 3-Methyladenine (3-MA) (Figure 5D, 5E). Autophagy is an adaptive mechanism that promotes survival in response to unfolded protein associated with cell degradation and the recovery of excess unfolded proteins and defective organelles. Endoplasmic reticulum stress activates autophagy in tumor cells and regulates the viability of cancer cells 33. In our previous work, it was determined that the radioresistance of NPC is related to HSPA5 expression. Immunohistochemical experiments showed that the expression of autophagy and HSPA5 increased simultaneously in NPC tissues, and the increase was more obvious in radioresistance (Figure 5F). In addition, cell immunofluorescence showed a positive correlation between HSPA5 expression and autophagy marker LC3B (Figure 5G). Corresponding immunofluorescence results showed that the expressions of HSPA5 and LC3B were increased in tumor tissues, especially in radioresistive tissues. More importantly, HSPA5 was positively correlated with LC3B expression. These results suggest a possible interaction between HSPA5 and LC3B (Figure 5H).

**Figure 6:**
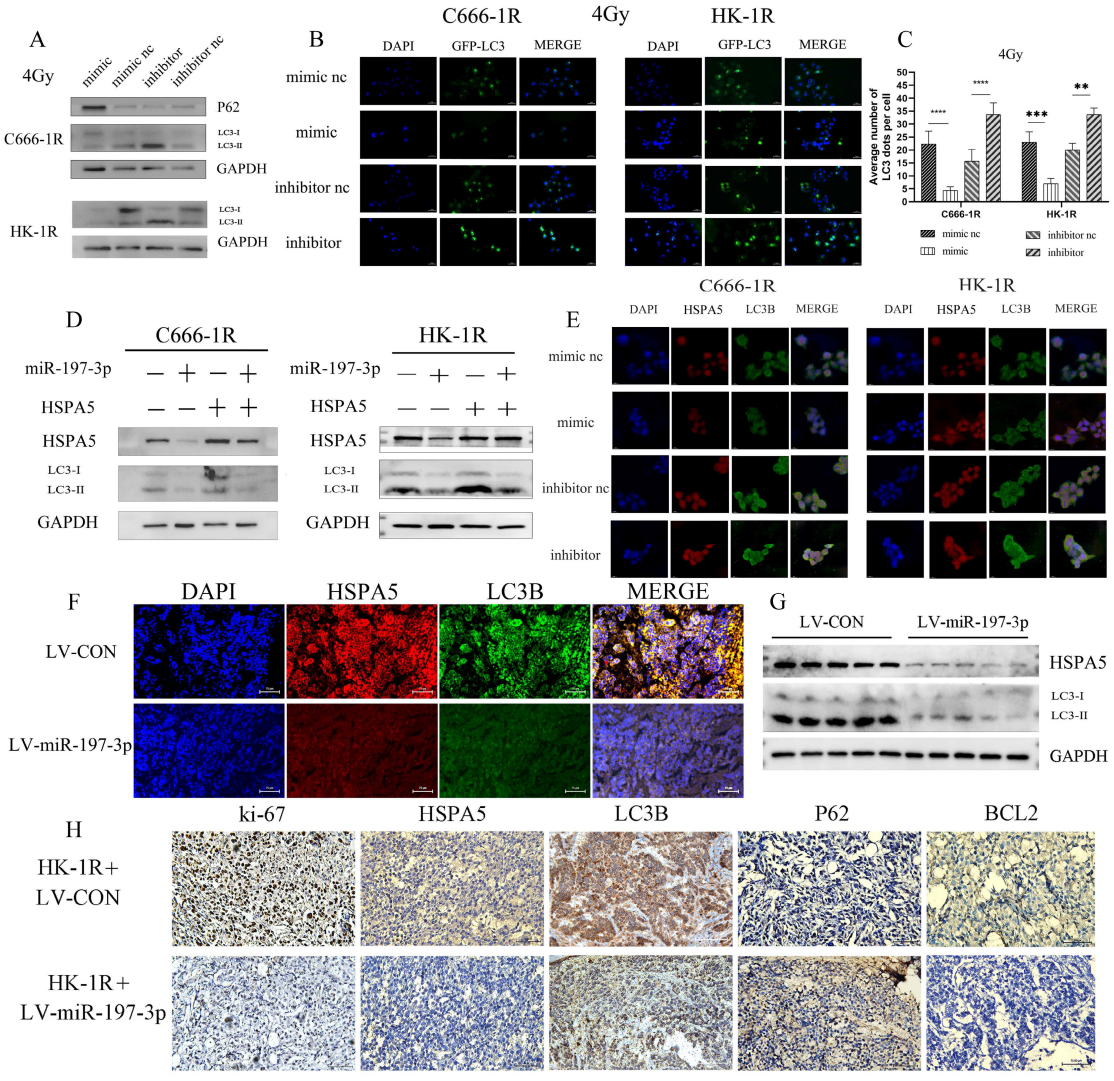
** MiR-197-3p downregulating HSPA5 modulates autophagy to control X-ray sensitivity. (A)** The expression of autophagy related proteins in transfected cells was detected by Western Blot. **(B, C)** The changes of autophagosomes after transfection with GFP-LC3 were detected. **(D)** Western Blot experiments verified that miR-197-3p regulated autophagy by targeting HSPA5. **(E)** The expression of HSPA5 and autophagy related protein LC3B in transfected cells was detected by cell immunofluorescence. **(F)** Immunofluorescence showed that tumors infected with LV-miR-197-3p expressed HSPA5 and LC3B in animal tissue. **(G)** Western Blot experiment showed the expression of LC3BI/LC3BII and HSPA5 in animal tumor protein. **(H)** Immunohistochemical results showed the expression of HSPA5, Ki-67, BCL2, LC3B and P62.

We investigated whether miR-197-3p could regulate autophagy in radioresistant NPC cells by targeting HSPA5. After transfection, NPC cells overexpressing miR-197-3p showed less autophagy under 4Gy of irradiation and increased autophagy after inhibition, which might be related to the radioresistance of NPC (Figure 6A) and corresponding changed in autophagosomes occurring after GFP-LC3 transfection (Figure 6B, 6C). Subsequently, Western Blot experiments were used to verify that miR-197-3p regulates autophagy by targeting HSPA5 (Figure 6D). After transfection of miR-197-3p in C666-1R and HK-1R cells, immunofluorescence revealed that miR-197-3p could inhibit the expression of HSPA5, and the protein LC3B related to autophagy in NPC cells, indicating that miR-197-3p could reduce HSPA5-related autophagy (Figure 6E). In animal experiments, immunofluorescence showed that tumors infected with LV-miR-197-3p expressed lower levels of HSPA5 and LC3B compared with strongly positive and highly expressed HSPA5 and LC3B in the control group (Figure 6F). Meanwhile, animal tumor protein Western Blot experiment showed a decrease in LC3BI/LC3BII, which further confirmed the lower expression of HSPA5 and autophagy in tumors after LV-miR-infection (Figure 6G). Immunohistochemical results showed that the expression of HSPA5, Ki-67 and BCL2 decreased, and the expression of autophagy marker LC3B decreased and the expression of P62 increased after LV-miR-197-3p infection (Figure 6H). These results suggest that miR-197-3p can target HSPA5 and inhibit tumor proliferation and anti-apoptosis and autophagy *in vivo*.

### Exosomal transfer of miR-197-3p inhibits NPC progression by AKT/mTOR pathway

**Figure 7:**
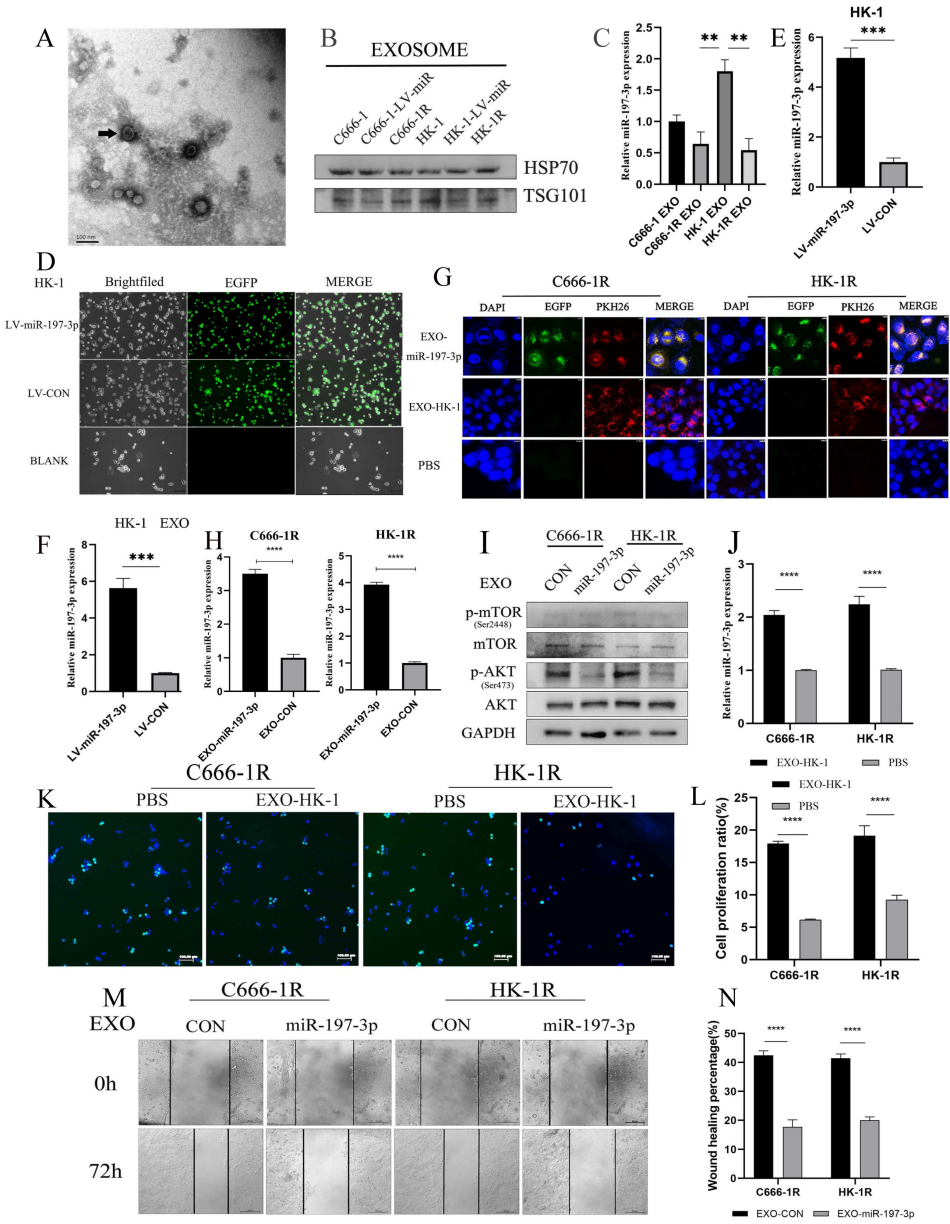
** Exosomal transfer of miR-197-3p inhibits NPC progression by AKT/mTOR pathway. (A)** Detection of NPC-EXOs by transmission electron microscopy. **(B)** Western Blot confirmed the exosome-related proteins (TSG101 and HSP70). **(C)** QRT-PCR was compared the exosomal miR-197-3p expression. **(D)** HK-1 with EGFP-labeled LV-miR-197-3p. **(E)** QRT-PCR tested the infection efficiency. **(F)** The expression of miR-197-3p in LV-HK-1-EXOs was detected by qRR-PCR. **(G)** PKH26 - labeled exosomes were ingested by radioresistant NPC. **(H)** QRT-PCR to detect changes in miR-197-3p levels after EXO-miR-197-3p and EXO-CON uptake in radioresistant NPC cells. **(I)** The AKT/mTOR pathway changes after exosomes ingested were detected by Western Blot. **(J)** The expression of miR-197-3p was detected by qRT-PCR after the incubation of EXO-HK-1. **(K, L)** CCK-8 assay was detected the proliferation after the incubation of EXO-HK-1. **(M, N)** The effect of EXO-miR-197-3p on migration was examined by Wound Healing assay.

Exosomes were extracted from supernatant of cell culture medium by supercentrifugation and SBI exosome kit. Exosomes were identified by transmission electron microscopy (Figure 7A). Western blotting further confirmed the abundant expression of exosome-related proteins, including TSG101 and HSP70 (Figure 7B). In this study, we chose NPC cell lines C666-1 and HK-1 as models to investigate tumor-derived exosomes and exosomal miRNAs, and the corresponding radioresistant NPC cell lines, C666-1R and HK-1R, were used as controls. We compared exosomal miR-197-3p expression among cell lines with qRT-PCR. We found that miR-197-3p was reduced in exosomes derived from radioresistant NPC cells in comparison to radiosensitive NPC cells (Figure 7C), which was consistent with the cellular miR-197-3p levels in NPC cells. Subsequently, we infected HK-1 with EGFP-labeled LV-miR-197-3p (Figure 7D) and tested the infection efficiency using qRT-PCR (Figure 7E). Meanwhile, in exosomes extracted from HK-1-LV-miR-197-3p cells, levels of exosomal miR-197-3p were much higher than those extracted from control cells (Figure 7F). Subsequently, exosomes were labelled with PKH26 and then co-cultured with C666-1R and HK-1R for 24 h. Confocal microscopy revealed that exosomes could be ingested by radioresistant NPC cells, and overexpressed miR-197-3p could be encapsulated and ingested by exosomes (Figure 7G). We used qRT-PCR to detect changes in miR-197-3p levels after EXO-miR-197-3p and EXO-CON uptake in radioresistant NPC cells. After ingestion of exosomes overexpressing miR-197-3p, expression was also increased in radioresistant NPC cells (Figure 7H). MiR-197-3p has been confirmed to directly target HSPA5 and to regulate the AKT pathway in previous literature. Changes in the AKT/mTOR pathway after uptake were detected by Western Blot, and the results were similar to those of miR-197-3p overexpressed in cells in the previous stage. After ingestion of EXO-miR-197-3p, the AKT/mTOR pathway was inhibited (Figure 7I). As lentivirus- carried exosomes were labelled with EGFP fluorescence, we also extracted HK-1 exosomes for co-culture experiments. It could be seen that the expression of miR-197-3p was also increased after the incubation of EXO-HK-1(Figure 7J). EDU assay was conducted to detect the proliferation efficiency, and it was found that the proliferation of the HK-1 exosome group was reduced in comparison to the PBS control group (Figure 7K, 7L). We speculate that exosomes secreted by radiotherapy-sensitive NPC cells can inhibit cell proliferation. Exploration of the role of EXO-miR-197-3p revealed that it inhibited the migration of NPC cells (Figure 7M, 7N). In summary, we can conclude that exosomal miR-197-3p play an important role in the progression of NPC.

### EXO-miR-197-3p regulates radioresistance and autophagy by targeting HSPA5

**Figure 8:**
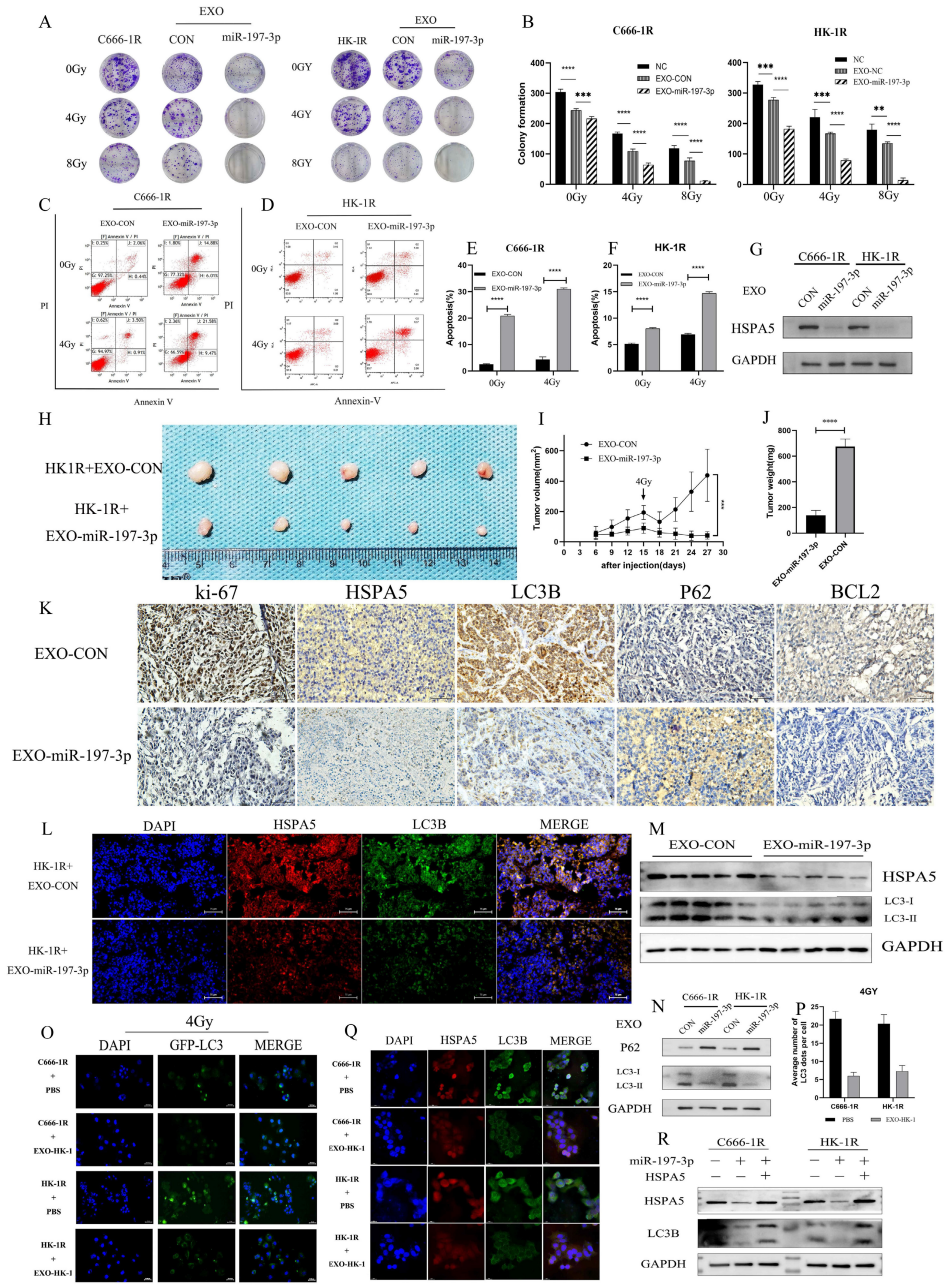
** EXO-miR-197-3p regulates radioresistance and autophagy by targeting HSPA5. (A, B)** Colony formation assay was detected the radioresistance of the cells ingested EXO-miR-197-3p. **(C, D, E, F)** Flow cytometry was detected apoptosis after uptake of exosomes at 4G_Y_. **(G)** Western Blot was detected the expression of HSPA5 after cell uptake of EXO-miR-197-3p. **(H)** Animal experiments tested the effect of EXO-miR-197-3p. **(I, J)** Tumor volume and weight were measured. **(K)** Immunohistochemical results showed that after EXOs-miR-197-3p was injected; the expressions of HSPA5, Ki-67, BCL2, LC3B and P62 were analyzed. **(L)** Immunohistofluorescence showed that the expression of HSPA5 and LC3B; Immunohistofluorescence showed that the expression of HSPA5 and LC3B was decreased in the EXO group. **(M)** Western Blot assay detected the expression of LC3B and HSPA5 in tumor. **(N)** Western Blot detected the expression of HSPA5 after cell uptake of EXO-miR-197-3p. **(O, P)** After transfection GFP-LC3, autophagosome formation was detected after EXO-HK-1 ingestion. **(Q)** The expressions of HSPA5 and autophagy related protein LC3B in cell uptake of EXO-HK-1 was detected by cell immunofluorescence. **(R)** The correlation between miR-197-3p, HSPA5 and LC3B was detected by Western Blot.)

**Figure 9:**
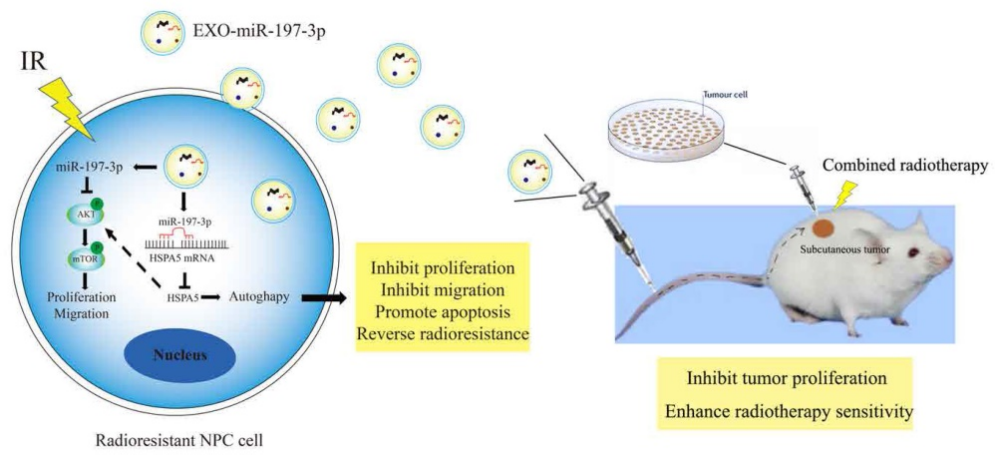
Mechanism diagrams of EXO-miR-197-3p regulating NPC radioresistance and autophagy by targeting HSPA5. EXO-miR-197-3p inhibits NPC progression (proliferation and migration) and radioresistance by regulating phosphorylation of AKT/mTOR signaling pathway and autophagy in recipient NPC cells and in vivo.

We have demonstrated that miR-197-3p can target HSPA5 and regulate autophagy and NPC progression. Furthermore, exosomal miR-197-3p can inhibit NPC proliferation and migration. We hypothesized that exosomal miR-197-3p could also inhibit autophagy and regulate radioresistance in NPC, and carried out a colony formation assay to verify this. It was shown that exosomes secreted by the radiotherapy-sensitive strain HK-1 can improve radioresistance in NPC cells, while NPC cells that ingested EXOs-miR-197-3p were more sensitive to radiation (Figure 8A, 8B). Flow cytometry was used to detect apoptosis after uptake of exosomes. The apoptosis of EXO-miR-197-3p cells increased after ingestion, and apoptosis was more significant under the effect of 4 Gy of irradiation (Figure 8C, 8D, 8E, 8F). In addition, exosomal miR-197-3p can also regulate the expression of HSPA5 (Figure 8G). We confirmed our hypothesis *in vivo*, as HK-1R cells were found to be subcutaneously tumorigenic. Exosomes were injected into the tail vein every 3 days, and the tumor volume was measured, with 4 Gy radiation administered on the 15th day after tumor formation. The results revealed that EXO-miR-197-3p can inhibit the growth of NPC cells and enhance their radiotherapy sensitivity *in vivo* (Figure 8H, 8I, 8J). Subsequently, we conducted a mechanistic evaluation of this process. Immunohistochemical results showed that after EXOs-miR-197-3p was injected, the expression of HSPA5, Ki-67 and BCL2 decreased, while the expression of autophagy marker LC3B decreased and the expression of P62 increased (Figure 8K) Immunohistofluorescence showed that the expression of HSPA5 and LC3B was decreased in the EXO group (Figure 8L). Meanwhile, the expression of autophagy related protein in tumor was detected by Western Blot assay (Figure 8M). Therefore, EXOs-miR-197-3p can inhibit HSPA5 expression and autophagy *in vivo*. Western blotting confirmed the role of exosomes in autophagy. Compared with EXO-CON group, EXO-miR-197-3p could inhibit autophagy of radioresistant NPC cells (Figure 8N). After ingestion of EXO-HK-1, autophagosome formation was increased, indicating that exosomes secreted by radiosensitive cells can alter the autophagy process of resistant cells (Figure 8O, 8P). Meanwhile, immunofluorescence showed that the expression of HSPA5 and autophagy in NPC cells decreased after EXO-HK-1 was ingested (Figure 8Q). HSPA5 can restore autophagy inhibited by EXOs-miR-197-3p, further verifying the mechanism of miR-197-3p (Figure 8R). In summary, our results suggest that exosomal miR-197-3p can regulate the AKT/mTOR pathway, affecting the progression of NPC, and can target HSPA5 to inhibit autophagy and improve the radiotherapy sensitivity of NPC (Figure 9).

## Discussion

Radiotherapy is the main treatment option for NPC, but radioresistance leads to relapse and makes NPC refractory. Radioresistance of NPC cells can lead to poor prognosis, including radiotherapy failure and the accumulation of toxic effects associated with large doses of ionising radiation. The radiation response of cancer cells is complex and mainly determined by their intrinsic repair ability, which is controlled at a genetic level.

Expression of several miRNAs is associated with the occurrence and development of various tumors 34, 35, and influence tumor progression. However, their role in NPC, especially the molecular mechanism of radiotherapy resistance, has not been systematically reported. In this study, we found that miR-197-3p not only significantly inhibited proliferation and migration of NPC cells, and promoted apoptosis of NPC cells, but also enhanced their radiosensitivity.

Abnormal signalling pathways lead to various pathological processes, including tumor occurrence and progression. It has been confirmed that the activation of PI3K/Akt, as well as other signalling pathways promoting the survival of NPC cells, can affect the occurrence, development, and prognosis of NPC by affecting cell cycle, proliferation, DNA damage repair, apoptosis, and other biological processes 36, 37. It is of potential clinical significance to study the regulation mechanism of the signal pathway in the treatment of NPC 38. MiR-197-3p can also inhibit the growth of NPC cells by regulating the pro-growth signalling pathway PI3K/AKT/mTOR, which is consistent with studies in other tumors 13. In addition, it also inhibits the migration of NPC cells, which may be associated with reduced invasion and advanced metastasis of NPC.

Autophagy is a common mechanism of programmed cell death wherein lysosomes degrade most cytoplasmic contents to facilitate resource recovery and utilisation. Abnormal autophagy is closely related to tumor growth, metabolism, and immune diseases. Autophagy is an important factor leading to radioresistance and progression of NPC. This study reveals the role played by miR-197-3p in radiation-related autophagy and reversal of radioresistance in NPC. We have confirmed that miR-197-3p can reverse radiotherapy resistance of NPC, using both *in vivo* and *in vitro* models. Meanwhile, overexpression of miR-197-3p reversed the radioresistant biological functions of HSPA5-mediated autophagy in NPC cells. These results suggest that miR-197-3p has a potential role as a tumor suppressor in NPC.

Exosomes have demonstrated potential as therapeutic agents and can be utilized in exosome-based nanoparticle drug delivery strategies for cancer therapy, where they can deliver chemotherapeutic drugs in a targeted manner. They can sheathe drugs, and show strong uptake and intracellular retention after processing, resulting in good cytotoxicity and cell killing activity against tumors 39, 40. In colon cancer, the combined delivery of miR-21i and 5-FU with engineered exosomes effectively reversed the drug resistance of 5-FU-resistant colon cancer cells, and significantly enhanced the individual cytotoxicity of these drugs 41. The use of exosomes containing AntagomiR-BART10-5p and AntagomiR-18a may preferentially inhibit angiogenesis and growth of NPC, providing new insights into clinical intervention and treatment strategies for this type of cancer 42. Exosomes enriched with radiosensitive miRNAs might have significant potential for treating radioresistant NPC. Exosomal miRNAs are widely involved in NPC cell proliferation, migration, invasion, neovascularization, radioresistance, and regulation of the tumor immune microenvironment through intercellular communication, which makes them valuable biomarkers for early diagnosis and treatment of NPC 43-46. Exosomes can also reverse NPC progression, to some extent.

Interestingly, our data demonstrated that the levels of miR-197-3p-loaded exosomes (EXO-miR-197-3p) were downregulated in the culture medium of NPC radioresistant cell lines. In this study, we demonstrated the important role of exosomes in NPC. Exosomal miR-197-3p not only inhibits the proliferation and migration of NPC, but also enhances radiotherapy sensitivity. *In vivo* experiments also provide a theoretical basis for subsequent clinical treatment. The combination of radiotherapy and exosomes may be an effective method to treat radioresistant NPC 47. The potential role of exosomes in the clinical treatment of NPC remains to be further explored. Our findings suggest that EXO-miR-197-3p might act as a potential radiosensitizer and therapeutic target for the treatment of radioresistant NPC. Thus, EXO-miR-197-3p may represent a viable avenue for treatment of relapse and refractory NPC.

## Conclusion

In summary, EXOs-miR-197-3p as a molecular biomarker is associated with the prognosis of NPC. EXOs-miR-197-3p inhibits the progression of NPC cells by inhibiting AKT/mTOR signaling pathway. Moreover, it can reverse radioresistance of NPC by inhibiting HSPA5-mediated autophagy, providing a potential option for clinical treatment. Our results enhance our understanding of the molecular mechanisms underlying NPC progression and radioresistance. Although, therapeutic exosome development is in the preclinical stages, exosomal miRNAs are a promising treatment option for NPC.

## Supplementary Material

Supplementary table.

## Figures and Tables

**Table 1:** The clinical characteristics of 40 patients with NPC and 21 benign nasopharyngeal masses

Characteristic	No. (%) of NPC patients	No. (%) of benign masses
**Total cases**	40 (100%)	21 (100%)
**Age Range**	13-72	8-67
**Gender**		
Male	25 (62.5%)	12 (57.1%)
Female	15 (37.5%)	9 (42.9%)
**Recurrence**		
No	20 (50%)	-
Yes	20 (50%)	-

**Table 2:** Difference of HSPA5 level between benign and malignant nasopharyngeal masses (N=61, *X*^2^ Test)

	N	Scores of immunohistochemical staining			*X* ^2^	p
		Weak positive/Negative (0-2)	Positive (3-4)	Strong positive (5-6)		
**Benign nasopharyngeal mass**	21	19	2	0	24.16	<0.0001
**NPC**	40	10	15	15		
**Radiosensitive NPC**	20	8	8	4	6.933	0.0312
**Radioresistant NPC**	20	2	7	11		

Using *X*^2^ test. NPC, Nasopharyngeal Carcinoma.

**Table 3:** Correlation between HSPA5 expression and Radioresistance in NPC (N=40, *X*^2^ Test)

Variables	N	HSPA5 Level		*p*
		Low (17)	High (23)	
**Gender**				
Male	25	11	14	0.804
Female	15	6	9	
**Age**				
≤45	11	5	6	0.815
>45	29	12	17	
**TNM stage**				
I-II	20	12	8	0.025
III-IV	20	5	15	
**Radiation response**				
Sensitivity	20	14	6	0.001
Resistance	20	3	17	

**Abbreviations:** NPC: nasopharyngeal carcinoma; EBV: Epstein-Barr virus; IR: Ionizing Radiation; OS: overall survival; miRNA, miR: microRNA; ER: endoplasmic reticulum; EMT: epithelial mesenchymal transformation; HSPA5: The heat shock protein family A member 5; IHC: Immunohistochemistry; CCK-8: Cell counting kit-8; qRT-PCR: Quantitative real-time PCR.

## Abbreviations

NPC: nasopharyngeal carcinoma
EBV: Epstein-Barr virus
IR: Ionizing Radiation
OS: overall survival
miRNA, miR: microRNA
ER: endoplasmic reticulum
EMT: epithelial mesenchymal transformation
HSPA5: The heat shock protein family A member 5
IHC: Immunohistochemistry
CCK-8: Cell counting kit-8
qRT-PCR: Quantitative real-time PCR
