# Supplementary material for: Radiosensitizer EXO-miR-197-3p Inhibits Nasopharyngeal Carcinoma Progression and Radioresistance by Regulating the AKT/mTOR Axis and HSPA5-mediated Autophagy
Source: Int J Biol Sci. 2022 Feb 21;18(5):1878–95. doi: 10.7150/ijbs.69934 (PMC8935226; doi:10.7150/ijbs.69934)
Supplement: Supplementary file 1 — Supplementary table. [file ijbsv18p1878s1.pdf]

Table.1. Clinical information of patients in the NPC study

Patient	Gender	Age	Epstein-Barr virus infection (Negative:0, Positive:1)	Metastasis (No:0; metastasis: 1)	Recurrence (No:1; recurrence: 2)
**1**	M	65	0	1	0
**2**	M	47	0	1	0
**3**	M	53	0	0	0
**4**	M	63	1	1	0
**5**	M	63	-	0	0
**6**	F	47	0	1	0
**7**	F	55	0	1	0
**8**	M	48	0	1	1
**9**	F	62	1	1	0
**10**	M	65	0	1	1
**11**	M	13	1	1	0
**12**	F	49	0	0	1
**13**	F	57	-	0	0
**14**	M	42	-	1	0
**15**	M	19	-	1	0
**16**	F	69	-	0	1
**17**	F	62	-	0	1
**18**	M	53	-	0	1
**19**	M	55	-	0	1
**20**	M	59	0	0	1
**21**	F	61	-	1	0
**22**	F	61	-	0	1
**23**	M	56	-	0	1
**24**	F	36	1	0	1
**25**	F	38	1	1	0
**26**	M	45	1	1	1
**27**	M	40	-	1	0
**28**	M	42	-	1	0
**29**	M	42	1	0	0
**30**	F	72	0	1	0
**31**	F	53	-	0	1
**32**	M	64	-	1	0
**33**	F	50	-	0	1
**34**	F	48	-	0	1
**35**	M	48	-	0	1
**36**	M	46	-	0	1
**37**	M	52	0	0	1
**38**	M	53	1	0	1
**39**	M	34	0	1	0
**40**	M	40	-	1	1
